# Beyond the Books: Role of Extracurricular Activities in Medical Students

**DOI:** 10.31729/jnma.8613

**Published:** 2024-06-30

**Authors:** Madhu Sudan Aryal

**Affiliations:** 1 Nepalese Army Institute of Health Sciences, Sanobharyang, Kathmandu, Nepal

**Keywords:** *extracurricular activities*, *medical students*, *research*

## Abstract

Extracurricular activities (ECAs) in the field of medicine can be research related and non-research related activities that allows students to develop skills to be a good doctor in the future. Building a curriculum vitae, becoming a team player, managing stress, building good communication skills and good academics highlights the value of extracurricular activities. Challenges like time constraints, and resource limitation hinders the participation which needs to be addressed to encourage the medical students to take part in extracurricular activities.

## INTRODUCTION

Extracurricular activities(ECAs) in the field of medical school can be research related activities such as volunteering in a research programs, publishing research papers and non-research related activities such as playing sports, arts, writing literature and following our own hobbies which allows the students to develop skills to be a good doctor in the future.^1^ Research related activities helps us to develop better analysis and improved research skills whereas non research related activities helps us to improve our physical and mental health and improve our academic performance.^1^ Regardless of the importance of extracurricular activities barriers like fear of loss in academic performance, lack of time management skills makes students to step aside from participating in the ECAs.^2^

## IMPORTANCE OF ECAS

**1. Building a curriculum vitae (CV)**

A CV is a document with our personal qualification which can outline the personal interest and activities that shows our ability, and experience for the competitive areas.^3^ In medical school we participate in various research programs, do research, write literature and get engaged in different sports, where we are rewarded with certificates and medals which open the doors to opportunities making us standout from others.

**2. Becoming a team player**

Participating in ECAs provides us with knowledge, discipline, and to work effectively in team. It enhances our decision-making abilities and improves interpersonal skills making us more adept at dealing various people and situations.^2^

**3. Stress management**

Medical students have hectic academic schedule with frequent internal assessments which demands more effort, releasing adrenaline and cortisol which leads to stress.^4^ To reduce the stress we can get engaged in physical activities, social interaction with peers, journal keeping, literature writing. Such activities release endorphins which helps to reduce the stress.^5^

**4. Communication skills**

Empathy and trust are building blocks of good doctor-patient relationship which is achieved through communication. A good communication ability is an acquired skill gathered through trial and errors of multiple interaction with people which is enhanced when we get involved in ECAs like public speaking, presentation, and coordination programs.^6^

**5. Better academic performance**

The academic success in medicine involves mastering textbook content but this is not the sole factor. ECAs play an important role in academics by enhancing practical skills and fostering professional development. Activities like playing sports and doing research teaches us the value of time, and hard work which later result in regular attendance, better examination results, and better analysis.^7^

## BARRIERS IN ECAS

**1. Taking ECAs with academics**

Medical school has a tight academic schedule. Covering the taught lesson and pre-lecturing the upcoming classes can be time consuming. Participating in various ECAs makes us tired and to recover well our body needs proper rest. But the pressure to cover the academics makes us worry to get involved in the ECAs and we hesitate to participate or leave without completing the task.^1^ So, dedication, determination and discipline can be the important factors for the management of ECAs with academics.

**2. Accessing resources for research**

Research requires a proper literature review and citations. Conducting thorough literature reviews and accessing articles often requires substantial funds, especially for studies involving large populations. Pay-per-view articles further limit access, compounding the financial constraints faced by students. Additionally, institutes often struggle to provide adequate funds to support research activities leading to barrier in doing research.^8^

**3. Absence of ECAs in curriculum**

Medical schools don't have ECAs in the curriculum. Students who have potential in ECAs hesitate to take part in various programs such as sports week conducted by the institute as they are less appreciated for their results. Many institutes just prefer the academic result which pulls the students out from ECAs.^1^

## WAY FORWARD

The importance of ECAs in medical students is noteworthy. Regardless of the importance of the ECAs addressing barriers to participation is crucial to fully get the benefits. Time constraints, and resource availability is the major concern, emphasizing the institutional support and inclusion of cocurricular activities in curriculum.
